# Gender and spatial variation of loneliness among adolescents in three South Asian countries: prevalence and its correlates

**DOI:** 10.1186/s12889-025-26069-7

**Published:** 2026-01-27

**Authors:** Md. Khalid Hasan, Helal Uddin, Tahmina Bintay Younos, Nur A Habiba Mukta

**Affiliations:** 1 Department of Human Development and Family Sciences, Texas Tech University, Lubbock, TX 79409 USA; 2 Institute of Disaster Management and Vulnerability Studies, University of Dhaka, Dhaka, 1000 Bangladesh; 3 Department of Sociology, East West University, Dhaka, 1212 Bangladesh; 4 South Asian Institute for Social Transformation (SAIST), Dhaka, 1205 Bangladesh; 5 Department of Geography and GIS, University of Cincinnati, Cincinnati, OH 45221 USA; 6 Department of Global Public Health, Karolinska Institute, Solna, 17177 Sweden; 7 Department of Medicine, University of Oviedo, Oviedo, 33006 Spain

**Keywords:** Adolescent, Loneliness, Gender difference, Spatial variation, Afghanistan, Bangladesh, Pakistan

## Abstract

**Background:**

Adolescence is a critical developmental stage in the life course, and loneliness during this period has been linked to various mental health issues, social challenges, and academic difficulties. Hence, the study aimed to identify gender- and spatial variations in loneliness and its correlates among in-school adolescents in three South Asian countries.

**Methods:**

We analyzed data from 7,903 adolescents using the latest Global School-based Student Health Survey (GSHS) datasets from Afghanistan, Bangladesh, and Pakistan. Multiple logistic regression models, adjusted for socio-demographic variables, were conducted using STATA 14.

**Results:**

The prevalence of loneliness among male respondents was 12.28% [95% CI: 11.3–13.3], and 18.1% [95% CI: 16.8–19.5] in females. The prevalence of loneliness was highest among Afghan adolescents (34.8%), followed by Pakistani (11.4%) and Bangladeshi adolescents (8.4%). For both genders (male and female), loneliness was higher among the adolescents who were school truants, bullied, involved in physical fights, and experienced physical attacks. In addition, both male and female adolescents with anxiety-induced sleep disturbance, current tobacco users, and suicidal ideation had significantly higher odds of loneliness than their counterparts. Moreover, several poor mental health conditions, such as anxiety-induced sleep disturbance, bullying, suicidal ideation, and suicide plan, were significantly associated with higher odds of loneliness among in-school adolescents in Afghanistan, Bangladesh, and Pakistan. Additionally, respondents who were involved in physical fights were more likely to report feeling lonely.

**Conclusion:**

We identified gender and spatial variations in adolescent loneliness across three South Asian countries, highlighting the need for gender-sensitive and region-specific interventions. Policies should prioritize promoting inclusive environments and addressing cultural and resource-based challenges. To design targeted interventions, further research is needed to explore the socio-economic, environmental, and behavioral factors that influence loneliness.

**Supplementary Information:**

The online version contains supplementary material available at 10.1186/s12889-025-26069-7.

## Introduction

Although loneliness is a stereotyped mental health problem specific to older people, it is also a crucial mental health condition for the younger group [1, 2]. Adolescence is an important transformation and developmental stage when adolescents experience multiple social changes, such as emotional instability and increased sensitivity to loneliness [3, 4]. In general, loneliness is an unpleasant experience and feeling of loneliness due to the lack of social relationships [5]. Loneliness is a subjective and qualitative term closely linked to mental satisfaction with the quality and quantity of social relationships [3, 6]. Moreover, loneliness is a feeling of loneliness more than once per week [7, 8]. Therefore, loneliness is considered a multidimensional phenomenon that adversely impacts adolescent health and well-being [9, 10].

Loneliness negatively impacts adolescents’ mental and physical health, as well as risk behaviors [3, 9, 11]. For example, loneliness is associated with depressive symptoms, anxiety, perceived stress, and low self-esteem [12, 13], suicidal ideation [11], and psychiatric morbidity [14]. In addition, longitudinal studies also reported that loneliness increases the risk of mortality and morbidity [3, 9]. Moreover, loneliness has been associated with several risk behaviors, such as alcohol use, illicit drug use, and smoking [14, 15]. Loneliness also increases the risk of being bullied [16], injured [17], aggression [18], sexual risk behaviors [14], and truancy [19].

Due to the adverse effects of loneliness on adolescents, loneliness is a crucial public health concern, which demands identifying risk factors associated with adolescent loneliness [20]. Recent studies across different countries have identified multiple risk factors for loneliness. For example, a study of school adolescents in Sub-Saharan countries reported that peer rejection or the absence of close friends significantly affected adolescents’ psychological health and well-being [21]. Similarly, loneliness is linked to suicidal ideation [22, 23], being bullied [24, 25], and social anxiety [26]. In addition, household factors, including poverty [12], inadequate parental support [26], insufficient parental warmth and intimacy [13], and conflicts with parents [27], are significantly associated with adolescent loneliness. Moreover, several health and well-being-related factors, including physical inactivity [28], inadequate physical activity in physical education classes [29], leisure-time sedentary behavior [30], insufficient intake of fruits and vegetables [31], and higher consumption of soft drinks [32], are also associated with loneliness among adolescents. Hence, the degree of loneliness has been shown to be influenced differently by socio-environmental, emotional, and physical health risk factors [25, 33, 34].

Although loneliness harms the health and well-being of adolescents, their perceived loneliness might be reported differently depending on their gender identity. However, it remains inconclusive and needs clarification. The majority of the studies found that female adolescents have a higher risk of feeling lonely [14, 15], while other cross-sectional studies documented that the gender difference is mixed [35, 36] and inconsistent [37] as well as some studies have found no gender differences [14]. For example, Russian female adolescents had a higher risk of loneliness than male adolescents [14]. Similarly, female adolescents in the USA and Asian countries (i.e., the Philippines) were more likely to report loneliness than male adolescents [14, 15]. Conversely, some studies showed that the prevalence of loneliness in Myanmar [24] and Morocco [38] was higher among boys; however, there was no difference in loneliness among male and female adolescents in Tanzania [39]. Therefore, male and female adolescents in different countries encounter loneliness disproportionally. This is also relevant in South Asia, where structural inequalities unduly impact females’ health outcomes.

A recent study using the GSHS data reported that the prevalence of loneliness among Afghan adolescents was 35%, 8.5% among Bangladeshi adolescents, and 11% among Pakistani adolescents [40]. However, most previous cross-sectional studies conducted in Bangladesh have focused on loneliness among university students [41, 42] and the adult population [42, 43] or on loneliness caused by the recent COVID-19 pandemic [44, 45]. Besides, in Afghanistan, few studies have focused on the issue of loneliness and documented higher loneliness among adolescents (e.g., 26% using the GSHS 2003–2018 data) [40, 46]. Similarly, some recent studies reported a higher level of loneliness in Pakistan due to media use or during the COVID-19 lockdown [47–50]. Hence, country-stratified analysis underscores the importance of drafting context-specific policy recommendations, as suggested interventions that are effective in one South Asian country may not be effective in another.

However, theoretically, this study adhered to Bronfenbrenner’s Ecological Systems Theory, which emphasizes the intricate relationships between individuals and their environments across multiple layers [51]. This theory helps explain the consequences of multi-level factors on adolescent loneliness, including the microsystem, mesosystem, macrosystem, exosystem, and chronosystem [52]. By applying this theoretical framework, our study analyzed how socio-demographic variables, gender norms, and spatial variations in three South Asian countries influence adolescents’ experiences of loneliness. In this context, the microsystem refers to direct influences, such as parental supervision and peer support, that significantly impact the formation of feelings of isolation or inclusion. Similarly, the mesosystem reflects the connection between these settings, such as how parental connectedness affects the quality of peer bonding. The exosystem indicates community and school-level factors, including school truancy, bullying, physical attacks, and passive smoking, which indirectly influence loneliness. Then, the macrosystem reflects broader cultural norms and structural settings, including health risk behaviors such as fast-food and soft drink consumption, smoking, and physical inactivity, as well as gender expectations. Lastly, the chronosystem indicates some transitional processes like poor mental health outcomes (e.g., suicidal ideation, anxiety, or having no close friends).

While global research on adolescent loneliness remains, few studies focus on South Asia, particularly Afghanistan, Pakistan, and Bangladesh, and do not focus on gender stratifying analysis, where there are persistent gender differences in access to health care utilization, resources, and health outcomes. Therefore, using national representative cross-sectional survey data, this study aimed to examine the prevalence of loneliness and its associated risk factors among male and female adolescents in three South Asian countries. Thus, detecting the gender and spatial stratified prevalence of loneliness and its risk factors might be an excellent initiative to understand the influence of gender and exposure to loneliness among adolescents in South Asia, which would be helpful for gender-sensitive adolescents’ mental health policy implications in those countries.

## Methods

### Data source and study design

We utilized cross-sectional datasets from the latest Global School-based Health Survey (GSHS), which is publicly available secondary data conducted in Afghanistan (2014), Bangladesh (2014), and Pakistan (2009). The GSHS is a nationally representative school-based survey developed by the WHO, UNICEF, UNESCO, UNAIDS, and CDC, among in-school students aged 13–17 years in low- and middle-income countries worldwide [53]. The GSHS comprises a comprehensive set of indicators, including demographics, mental health, violence, unintentional injury, alcohol and drug use, hygiene behaviors, and physical activity [54].

### Study settings

Three South Asian countries, Afghanistan, Bangladesh, and Pakistan, served as the settings of the study.

### Sampling and data collection

The GSHS employed a two-stage cluster sampling method to obtain a representative sample of in-school adolescents in each country. In the first stage, schools were selected proportionate to the total enrolment size in each country [53]. Then, classes were chosen randomly, and all students were invited to participate in the survey. A total of 2,579 student data were collected in Afghanistan GSHS, 2,989 in Bangladesh GSHS, and 5,129 in Pakistan GSHS.

For this study, we followed a complete-case approach. After listwise deletion of respondents with missing values on the included covariates or outcomes, 7903 adolescents were retained as the final analytical sample. Approximately 27% of students were excluded due to missing values on one or more variables. We acknowledge that this may introduce bias if the missingness is not entirely at random.

### Measures

#### Outcome variable

Loneliness was the outcome variable in this study. A single-item question, “During the last 12 months, how often have you felt lonely?” The response option to this question was 1 = never to 5 = always (coded 1–3 = 0, indicating “Not having loneliness” and 4–5 = 1, indicating “Having loneliness”) [39, 40, 55] (see Appendix, Table A1).

#### Predictor variables

Three predictor factors were included in the study. They included poor mental health factors (four variables), social–environmental factors (six variables), and health risk behavior factors (seven variables) (see Appendix, Table A1).

#### Control variables

Six variables — age, gender, country, hunger, peer support, and parental support — were included in the analyses as control variables. We included these variables based on their availability in the data and on recommendations from previous studies on GSHS. A detailed description of the control variables, including the predictor and outcome variables, is presented in the Appendix, Table A1.

Following Bronfenbrenner’s Ecological Systems Theory, this study mapped variables across ecological domains to indicate conceptual alignment. Microsystem variables include peer and parental factors (e.g., peer support, parental supervision, connectedness, bonding) and immediate social relationships (e.g., having no close friends). Mesosystem variables included school truancy and experiences of bullying, physical attacks, or fights. Exosystem variables captured indirect environmental influences such as exposure to passive smoking, health risk behaviors, and dietary and activity-related behaviors shaped by family or community contexts. Finally, chronosystem variables included temporal or developmental variables like age, hunger, and mental health trajectories (anxiety, suicide ideation, suicide plan).

### Data analysis

Descriptive and inferential statistical analyses were performed using Stata version 14.0 (Stata Corporation, 133 College Station, Texas, USA). The prevalence of loneliness, adjusted for control variables, is reported in Table 1. Moreover, the gender and spatial variation in the prevalence of loneliness, as explained by the explanatory variables, was assessed using the chi-square test in Table 2. Then, multiple logistic regressions (MLR) were used to estimate the associations (AOR, 95% CI) between gender-segregated loneliness and poor psychological health factors, social-environmental factors, and health risk behavior factors (Table 3). Additionally, Table 4 presents spatial differences in the associations (AOR, 95% CI) between loneliness and explanatory variables. The multicollinearity of MLR models was checked using the Pearson correlation matrix, variance inflation factor (VIF), and tolerance statistics. No unacceptable collinearity issues were found in the regression analyses. Missing values were excluded from the statistical analysis. Statistical significance was set at a p-value of ≤ 0.05. All analyses applied the survey weights provided in the GSHS dataset to adjust for the two-stage cluster sampling design and non-response, thereby confirming nationally representative estimates of in-school adolescents in each country. The complex survey design of the GSHS was specified in STATA software using the *psu*,* strata*,* singleunit* commands to include the primary sampling unit, student weights and stratification. Then, all analytic models were estimated applying the *svy* command, indicating standard errors were adjusted for stratification and clustering. Data was downloaded and analyzed between March and May 2023.

**Table 1:** Sample characteristics and control variables among adolescents in three countries of South Asia (*n* = 7903)

Variables (Control)	Sample	Loneliness	
	*n* (%)	% (95% CI)	AOR (95% CI)
All	7903 (100)	14.73 (14.0–15.5)	**–**
Age in years			
13 or less	1588 (20.09)	10.96 (9.5–12.6)	Ref.
14–15	5412 (68.48)	13.21 (12.3–14.1)	1.31 (0.88–1.95)
16 or more	903 (11.43)	30.45 (27.5–33.6)	2.09 (1.18–3.70)**
Gender			
Female	3326 (42.09)	18.1 (16.8–19.5)	Ref.
Male	4577 (57.91)	12.28 (11.3–13.3)	0.95 (0.73–1.23)
Country			
Afghanistan	1444 (18.27)	34.76 (32.3–37.3)	Ref.
Bangladesh	2433 (30.79)	8.38 (7.3–9.6)	0.22 (0.14–0.35)***
Pakistan	4026 (50.94)	11.38 (10.4–12.4)	0.26 (0.18–0.38)***
Hungry			
Never	4669 (59.08)	13.77 (12.8–14.8)	Ref.
Rarely/sometimes	2403 (30.41)	13.73 (12.4–15.2)	0.67 (0.49–0.92)**
Mostly/always	831 (10.51)	22.98 (20.2–26.0)	1.21 (0.80–1.83)
Peer support			
Never/rarely	1947 (24.64)	14.79 (13.2–16.4)	Ref.
Sometimes	1791 (22.66)	13.18 (11.6–14.8)	0.89 (0.65–1.21)
Mostly/always	4165 (52.70)	15.37 (14.3–16.5)	0.74 (0.54–1.00)
Parental support			
Low	3813 (48.25)	14.63 (13.5–15.8)	Ref.
Medium	1831 (23.17)	15.57 (13.9–17.3)	1.18 (0.83–1.68)
High	2259 (28.58)	14.21 (12.8–15.7)	0.84 (0.58–1.21)

***P ≤ 0.001; **P ≤ 0.01; *P ≤ 0.05; *Ref*  Reference category, *AOR* Adjusted Odds Ratio

**Table 2:** Characteristics of explanatory variables and prevalence of loneliness by gender and country (*n* = 7903)

	Sample Characteristics	Loneliness (prevalence)							
Variables	Total, *n* (%)	Weighted prevalence	Gender			Country			
		% (95% CI)	Girls (%)	Boys (%)	*p* values	Afghanistan (%)	Bangladesh (%)	Pakistan (%)	*p* values
*Poor mental health factors*									
No close friends									
Yes	617 (7.81)	22.9 (19.6–26.4)	9.68	6.45	0.001***	9.07	8.22	7.10	0.038*
No	7286 (92.19)	14.0 (13.3–14.9)	90.32	93.55		90.93	91.78	92.90	
Anxiety–induced sleep disturbance									
Yes	755 (9.55)	52.3 (48.7–55.9)	12.09	7.71	0.001***	25.0	3.62	7.60	0.001***
No	7148 (90.45)	10.8 (10.0–11.5)	87.91	92.29		75.0	96.38	92.40	
Suicidal ideation									
Yes	574 (7.26)	34.7 (30.8–38.7)	7.52	7.08	0.459	14.20	4.36	6.53	0.001***
No	7329 (92.74)	13.2 (12.4–14.0)	92.48	92.92		85.80	95.64	93.47	
Suicide plan									
Yes	597 (7.55)	33.0 (29.2–36.9)	7.91	7.30	0.311	12.81	5.38	6.98	0.001***
No	7306 (92.45)	13.2 (12.5–14.0)	92.09	92.7		87.19	94.62	93.02	
*Social–environmental factors*									
Bullied in past month									
Yes	2731 (34.56)	22.9 (21.3–24.5)	27.81	39.46	0.001***	39.13	19.28	42.15	0.001***
No	5172 (65.44)	10.4 (9.6–11.3)	72.19	60.54		60.87	80.72	57.85	
Physically attacked in past year									
Yes	3120 (39.48)	17.1 (15.8–18.5)	36.23	41.84	0.001***	28.67	54.21	34.45	0.001***
No	4783 (60.52)	13.2 (12.2–14.2)	63.77	58.16		71.33	45.79	65.55	
In physical fight in past year									
Yes	2443 (30.91)	18.3 (16.8–19.9)	17.29	40.81	0.001***	33.03	14.18	40.26	0.001***
No	5460 (69.09)	13.1 (12.2–14.1)	82.71	59.19		66.97	85.82	59.74	
Passive smoking in past week									
Yes	3627 (45.89)	17.3 (16.0–18.5)	29.56	57.77	0.001***	47.71	32.18	53.53	0.001***
No	4276 (54.11)	12.6 (11.6–13.6)	70.44	42.23		52.29	67.82	46.47	
School truancy (past month)									
Yes	5844 (73.95)	15.6 (14.7–16.6)	79.89	69.63	0.001***	84.90	64.45	75.76	0.001***
No	2059 (26.05)	12.2 (10.8–13.7)	20.11	30.37		15.10	35.55	24.24	
*Health risk behaviors factors*									
Current smoking									
Yes	514 (6.5)	18.7 (15.4–22.3)	1.11	10.42	0.001***	4.50	7.07	6.88	0.003**
No	7389 (93.5)	14.5 (13.7–15.3)	98.89	89.58		95.5	92.93	93.12	
Current tobacco use									
Yes	461 (5.83)	18.9 (15.4–22.7)	1.29	9.13	0.001***	2.91	6.37	6.56	0.001***
No	7442 (94.17)	14.5 (13.7–15.3)	98.71	90.87		97.09	93.63	93.44	
Physical inactivity									
Yes	1883 (23.83)	11.5 (10.1–13.1)	26.46	21.91	0.001***	12.33	49.77	12.27	0.001***
No	6020 (76.17)	15.7 (14.8–16.7)	73.54	78.09		87.67	50.23	87.73	
Leisure time sedentary behavior (≥ 3 h/day)									
Yes	934 (11.82)	22.8 (20.1–25.6)	12.72	11.16	0.035*	22.02	13.52	7.13	0.001***
No	6969 (88.18)	13.6 (12.8–14.5)	87.28	88.84		77.98	86.48	92.87	
Fast food consumption (≥ 2 days/week)									
Yes	1750 (22.14)	16.1 (14.4–17.9)	24.8	20.21	0.001***	33.66	41.06	6.58	0.001***
No	6153 (77.86)	14.3 (13.5–15.2)	75.2	79.79		66.34	58.94	93.42	
Soft drink consumption (≥ 3 drinks/day)									
Yes	517 (6.54)	17.4 (14.2–21.0)	8.66	5.0	0.001***	7.13	12.66	2.63	0.001***
No	7386 (93.46)	14.5 (13.7–15.4)	91.34	95.0		92.87	87.34	97.37	
Inadequate fruit intake									
Yes	1026 (12.98)	15.7 (13.5–18.1)	10.97	14.44	0.001***	11.01	3.66	19.32	0.001***
No	6877 (87.02)	14.6 (13.8–15.4)	89.03	85.56		88.99	96.34	80.68	
Inadequate vegetable intake									
Yes	953 (12.06)	14.5 (12.3–16.9)	6.52	16.08	0.001***	8.24	0.90	20.17	0.001***
No	6950 (87.94)	14.8 (14.0–15.6)	93.48	83.92		91.76	99.10	79.83	

***P ≤ 0.001; **P ≤ 0.01; *P ≤ 0.05

**Table 3:** Associations between loneliness and explanatory variables among boys and girls in three South Asian countries

Variables	Girls (*n* = 3326) AOR (95% CI)^+^	Boys (*n* = 4577) AOR (95% CI)^+^	Both Sex (*n* = 7903) AOR (95% CI)
Poor mental health factors			
No close friends (Yes)^¥^	1.63 (1.06–2.50)**	2.60 (1.55–4.34)***	2.03 (1.37–3.00)***
Anxiety–induced sleep disturbance (Yes)^¥^	6.11 (4.09–9.11)***	8.64 (5.90–12.66)***	7.47 (5.54–10.07)***
Suicidal ideation (Yes)^¥^	2.60 (1.56–4.35)***	3.18 (1.80–5.64)***	2.93 (1.93–4.47)***
Suicide plan (Yes)^¥^	2.66 (1.82–3.89)***	2.55 (1.48–4.40)***	2.57 (1.69–3.91)***
Social–environmental factors			
Bullied in past month (Yes) ^¥^	1.94 (1.43–2.63)***	3.24 (2.03–5.18)***	2.75 (1.98–3.82)***
Physically attacked in past year (Yes) ^¥^	1.82 (1.32–2.51)***	1.70 (1.06–2.73)**	1.73 (1.25–2.40)***
In physical fight in past year (Yes) ^¥^	1.08 (0.82–1.44)	2.53 (1.77–3.62)***	2.01 (1.51–2.67)***
Passive smoking in past week (Yes) ^¥^	1.92 (1.42–2.58)***	1.29 (0.83–2.03)	1.47 (1.03–2.09)**
School truancy (past month) (Yes) ^¥^	1.28 (0.93–1.76)	1.02 (0.67–1.57)	1.11 (0.79–1.56)
Health risk behaviors factors			
Current smoking (Yes) ^¥^	4.67 (1.23–17.76)**	1.71 (0.76–3.85)	1.87 (0.87–4.02)
Current tobacco use (Yes) ^¥^	4.65 (1.32–16.39)**	3.57 (1.71–7.45)***	3.58 (1.73–7.38)***
Physical inactivity (Yes) ^¥^	0.91 (0.63–1.32)	1.29 (0.80–2.08)	1.12 (0.79–1.60)
Leisure time sedentary behavior/day (≥ 3 h/day) (Yes) ^¥^	2.05 (1.47–2.85)***	1.20 (0.73–1.98)	1.45 (1.00–2.11)**
Fast food consumption (≥ 2 days/week) (Yes) ^¥^	1.15 (0.84–1.58)	1.61 (0.91–2.84)	1.43 (0.91–2.26)
Soft drink consumption (≥ 3 drinks/day) (Yes) ^¥^	0.99 (0.56–1.77)	1.70 (0.90–3.21)	1.49 (0.90–2.46)
Inadequate fruit intake (Yes) ^¥^	1.02 (0.72–1.42)	1.26 (0.67–2.36)	1.15 (0.75–1.75)
Inadequate vegetable intake (Yes) ^¥^	0.88 (0.68–1.13)	1.14 (0.69–1.88)	1.05 (0.75–1.47)

***P ≤ 0.001; **P ≤ 0.01; *P ≤ 0.05; *AOR* Adjusted Odds Ratio, *CI* Confidence Interval; ¥ indicates the reference category is No

^+^Each outcome is entered in a separate model controlling for age, country, hunger, peer support & parental support

**Table 4:** Country–wise associations between loneliness and explanatory variables among adolescents in three South Asian countries

Variables	Afghanistan AOR (95% CI)^+^	Bangladesh AOR (95% CI)^+^	Pakistan AOR (95% CI)^+^
Poor mental health factors			
No close friends (Yes) ^¥^	0.95 (0.53–1.73)	2.28 (1.26–4.13)***	2.24 (1.37–3.67)***
Anxiety–induced sleep disturbance (Yes) ^¥^	3.84 (2.80–5.25)***	7.71 (4.16–14.27)***	8.04 (5.36–12.07)***
Suicidal ideation (Yes) ^¥^	2.91 (2.02–4.18)***	3.38 (1.57–7.27)***	2.46 (1.65–3.65)***
Suicide plan (Yes) ^¥^	2.53 (1.61–3.99)***	2.47 (1.17–5.22)**	2.74 (1.85–4.03)***
Social–environmental factors			
Bullied in past month (Yes) ^¥^	2.95 (2.20–3.95)***	3.03 (1.73–5.30)***	2.01 (1.56–2.61)***
Physically attacked in past year (Yes) ^¥^	2.02 (1.40–2.94)***	1.64 (0.91–2.95)	1.59 (1.24–2.03)***
In physical fight in past year (Yes) ^¥^	1.34 (1.11–1.61)***	2.52 (1.62–3.91)***	1.43 (1.05–1.95)**
Passive smoking in past week (Yes) ^¥^	1.60 (1.14–2.24)***	1.40 (0.79–2.49)	1.64 (1.33–2.03)***
School truancy (past month) (Yes) ^¥^	0.67 (0.38–1.17)	1.09 (0.63–1.90)	1.05 (0.85–1.31)
Health risk behaviors factors			
Current smoking (Yes) ^¥^	1.68 (0.72–3.91)	1.56 (0.46–5.31)	2.84 (1.76–4.60)***
Current tobacco use (Yes) ^¥^	2.15 (0.57–8.16)	5.61 (1.94–16.22)***	2.62 (1.63–4.24)***
Physical inactivity (Yes) ^¥^	2.07 (1.18–3.62)**	1.06 (0.66–1.69	1.55 (0.99–2.45)
Leisure time sedentary behavior (≥ 3 h/day) (Yes) ^¥^	0.86 (0.53–1.39)	1.34 (0.73–2.43)	2.03 (1.49–2.78)***
Fast food consumption (≥ 2 days/week) (Yes) ^¥^	0.91 (0.64–1.29)	1.59 (0.86–2.93)	1.46 (0.95–2.25)
Soft drink consumption (≥ 3 drinks/day) (Yes) ^¥^	1.08 (0.61–1.93)	1.52 (0.84–2.74)	1.42 (0.77–2.62
Inadequate fruit intake (Yes) ^¥^	1.42 (0.82–2.47	1.98 (0.88–4.44)	0.86 (0.64–1.15)
Inadequate vegetable intake (Yes) ^¥^	1.57 (0.94–2.64)	2.17 (0.44–10.71)	0.91 (0.70–1.19)

***P ≤ 0.001; **P ≤ 0.01; *P ≤ 0.05; *AOR* Adjusted Odds Ratio, *CI* Confidence Interval; ¥ indicates the reference category is No

^+^Each outcome is entered in a separate model controlling for age, country, hunger, peer support & parental support

### Ethical consideration

The World Health Organization’s Ethical Committee, along with respective governmental departments or ministries, granted ethical approval for the survey. In addition, the datasets were made freely available for future use. Informed consent was obtained from the respondents at the time of the survey as well as consent was also taken from the student’s parent or legal guardian [39]. Participation in the survey was voluntary. Hence, this study analyzed publicly available, de-identified data from the GSHS, which had received ethical approval from the relevant institutional review boards and ministries in each participating country. No additional ethical approval was required for this secondary analysis.

## Results

### Characteristics of the respondents and prevalence of loneliness

Among 7,903 respondents, 58% were male, 68% were aged 14–15, and half were from Pakistan (Table 1). A tenth reported experiencing frequent hunger due to food scarcity, and 48% reported having low parental support. Additionally, 8% lacked a close friend, 9.5% had anxiety-induced sleep disturbances, and 7.5% had suicide plans. Bullying affected 34.5%, physical attacks 39.4%, and 6.5% were current smokers (Table 2). Overall, 15% of adolescents reported loneliness. Hunger was associated with 21% higher odds of loneliness, although this association was not statistically significant. Adolescents aged 16 or older had 2.09 times higher odds of loneliness than those aged 13 or younger. Loneliness prevalence was 52.3% among those with anxiety-induced sleep disturbances, 34.7% among those with suicide ideation, and 23% among those with no close friends, frequent hunger, or excessive leisure time (Table 2).

### Gender and spatial variations of loneliness

Overall, male respondents had 5% [AOR = 0.95; 95% CI: 0.73–1.23] lower odds of loneliness than their female counterparts; however, this relationship was not statistically significant (Table 1). Among the female respondents, the prevalence of loneliness was 80% who were school truants, 36% who had been physically attacked in the past year, 28% who had experienced bullying in the past month, 26.5% who were physically inactive, and 17% who had experienced physical fights in the past year (Table 2). On the contrary, the highest prevalence of loneliness among the male respondents was 70% who had school truancy in the past month, followed by those who experienced passive smoking (58%), physical attacks (41%), physical fights (41%), and bullying (39.5%) (Table 2).

The prevalence of loneliness among Afghan adolescents was highest (34.8%), followed by Pakistani (11.4%) and Bangladeshi adolescents (8.4%). The highest prevalence of loneliness was observed among school truants across all three countries: Afghanistan (84.9%), Bangladesh (64.55%), and Pakistan (75.8%). In Afghanistan, the prevalence of loneliness was 39% among the respondents who experienced bullying in the past month, 33.7% who ate fast food (≥ 2 days/week), and 33% who participated in physical fights in the past year. In Bangladesh, the prevalence of loneliness was high among the respondents who were physically attacked in the past year (54%), physically inactive (50%), bullied in the past month (42%), and consumed fast food ≥ 2 days a week (41%). In Pakistan, in-school adolescent respondents who were bullied, involved in physical fights, and experienced physical attacks had a higher prevalence rate of loneliness. 42%, 40%, and 34.5%, respectively (Table 2).

### Gender differences in the associations of loneliness with explanatory variables

Table 3 represents the adjusted odds ratios from logistic regressions that examined the association between loneliness and explanatory variables after controlling for age, country, hunger, peer support, and parental support variables. For both sexes, respondents having anxiety-induced sleep disturbance [AOR = 7.47; 95% CI: 5.54–10.07], current tobacco users [AOR = 3.58; 95% CI: 1.73–7.38], having suicidal ideation [AOR = 2.93; 95% CI: 1.93–4.47], and having suicide plan [AOR = 2.57; 95% CI: 1.69–3.91] had statistically significant higher odds of loneliness than their counterparts.

Among the female respondents, adolescents having anxiety-induced sleep disturbance [AOR = 6.11; 95% CI: 4.09–9.11], current smokers user [AOR = 4.67; 95% CI: 1.23–17.76], current tobacco users [AOR = 4.65; 95% CI: 1.32–16.39], having suicidal plan [AOR = 2.66; 95% CI: 1.82–3.89], and having suicide ideation [AOR = 2.60; 95% CI: 1.56–4.35] had statistically significant higher odds of loneliness than their counterparts. Similarly, among the male respondents, adolescents having anxiety-induced sleep disturbance [AOR = 8.64; 95% CI: 5.90–12.66], current tobacco users [AOR = 3.57; 95% CI: 1.71–7.45], having suicidal ideation [AOR = 3.18; 95% CI: 1.80–5.64], and having suicide plan [AOR = 2.55; 95% CI: 1.48–4.40] had statistically significantly greater odds of loneliness than their counterparts (Table 3).

### Spatial differences in the associations of loneliness with explanatory variables

In Afghanistan, several poor mental health factors, such as anxiety-induced sleep disturbance [AOR = 3.84; 95% CI: 2.80–5.25], bullied in the past month [AOR = 2.95; 95% CI: 2.20–3.95], suicidal ideation [AOR = 2.91; 95% CI: 2.02–4.18], and having suicide plan [AOR = 2.53; 95% CI: 1.61–3.99] were significantly associated with higher odds of loneliness among the in-school adolescents than their counterparts. Adolescents who reported a suicide plan had considerably higher odds of loneliness (AOR = 2.53; 95% CI: 1.61–3.99) compared with their counterparts.

These three factors were also significantly associated with the loneliness of the respondents in Bangladesh and Pakistan (Table 4). In addition, current tobacco use was also significantly associated with the loneliness of the respondents in Bangladesh [AOR = 5.61; 95% CI: 1.94–16.22] and Pakistan [AOR = 2.62; 95% CI: 1.63–4.24]. Moreover, respondents with no close friends had significantly higher odds of loneliness than their counterparts in Bangladesh [AOR = 2.28; 95% CI: 1.26–4.13] and Pakistan [AOR = 2.24; 95% CI: 1.37–3.67]. Leisure-time sedentary behavior (≥ 3 h/day) was also significantly associated with the risk of loneliness among Pakistani adolescents [AOR = 2.03; 95% CI: 1.49–2.78] (Table 4).

## Discussion

To our knowledge, this is the first study to investigate gender-stratified adolescent loneliness in these three South Asian countries and explore the associated risk factors. Our analysis revealed an overall prevalence of adolescent loneliness of 14.7%. The prevalence of loneliness among girls (18.1%) was higher than that of boys (12.3%). The highest prevalence of adolescent loneliness was found in Afghanistan (34.8%), followed by Pakistan (11.4%) and Bangladesh (8.4%). However, the overall prevalence reported in this study was higher than in Southeast Asian countries in 2007 and 2013 using the GSHS data (7.8%) [24] but lower than in four Caribbean countries in 2016-17 (15.3%) [19], Nigeria in 2017 (25.8%) [56], Tanzania in 2017 (17.4%) [39], Ghana in 2012 (18.1%) [57], Morocco in 2016 (19.8%) [38], and 25 countries in the Americas in 2018 (18.1%) [58].

Afghanistan has experienced armed conflict, social injustice, widespread poverty, broken education, and health care management, as well as disrupted community networks [59–61]. Therefore, several crucial factors, including prolonged periods of conflict, poverty, disrupted family relations, exposure to various forms of violence, weakened social support, and disrupted social structures, contribute to the higher prevalence of loneliness in Afghanistan [61, 62]. For example, among 300 children from Kabul, 90% reported that they could die due to war, and 80% marked themselves as frightened, sad, and incapable of coping with life [63]. Consequently, children are more vulnerable to trauma due to the violence, resulting in short and long-term mental consequences [64]. In addition to violence, Afghan children and adolescents face multiple forms of violations of rights, such as corporal punishment, forced marriage, and hazardous working conditions [65, 66].

In line with previous studies, the findings showed that adolescent girls had a higher likelihood of loneliness than boys [24, 38, 39]. For example, adolescent girls reported higher perceived loneliness than boys in the USA (14.7% vs. 6.7%), Russia (14.4% vs. 8.9%), using the Social and Health Assessment (SAHA) data in 2003 [14], Tanzania using 2017 GSHS data (51% vs. 49%) [39], four Caribbean countries using 2016–17 GSHS data (50% vs. 49%) [19], and six countries in Southeast Asia using 2013-17 GSHS data [24]. It may happen due to differences in girls’ coping strategies to face stressors compared to boys, which makes girls more vulnerable to loneliness [67, 68]. Although loneliness and psychological distress are both interconnected, loneliness is a precursor to psychological distress, while psychological distress can intensify feelings of loneliness as a result of departure from social interactions and difficulties in forming supportive relationships [69]. As a result, the interlink between these constructs builds a reinforcing cycle, specifically in girls who might have less effective coping mechanisms to alleviate stressors [70]. Although a small number of cross-sectional studies, such as in Myanmar [24] and Morocco [38], reported that the prevalence of loneliness was higher among boys, while many pieces of literature admit that girls are more vulnerable to loneliness using the same GSHS data [14, 19, 39], which has also been found among adolescent girls in three South Asian countries in our study.

In agreement with previous studies, our study found that several poor mental health factors: anxiety-induced sleep disturbance, suicidal ideation, making suicide plan, and having no close friends were strongly associated with adolescent loneliness [13, 22–24]. Having close friends is a vital protective factor in combating adolescent loneliness, highlighting the importance of close friends for social support and interaction [13]. Moreover, anxiety-generating thoughts, which hamper relaxation and sleep quality, are closely related to loneliness [3, 71]. Similarly, several cross-sectional studies using the GSHS data reported suicidal ideation or behavior as a risk factor for adolescent loneliness and depressive symptoms [22, 23, 72]. Although depression and loneliness are separate concepts, they may happen together and even be reciprocal [73, 74].

Several socio-environmental factors, such as bullying, physical fighting, physical attacks, frequent exposure to passive smoking, and hunger, were associated with adolescent loneliness. These findings were similar to those of previous studies conducted in ASEAN countries, Morocco, Caribbean countries, Spain, and the UK [19, 24, 34, 38, 75]. Adolescents with interpersonal victimization experience may worry about further victimization, which influences poor friendship formation, leading to loneliness [13, 76]. Moreover, adolescents with bullying experience possess poorer self-esteem, resulting in loneliness [77]. Finally, a study using nationally representative data on Kuwaiti school adolescents reported that physically fighting with fellow students was more likely to be rejected by peers; this might make adolescents isolated and lonely [78]. Therefore, adolescents who experience victimization were more likely to report avoidance and revenge, as well as higher odds of loneliness [75].

Similar to previous studies, our analysis revealed that health risk behavior factors, including current smoking, tobacco use, leisure-time sedentary behaviors, and physical inactivity- were associated with higher odds of loneliness among adolescents [19, 28, 30, 39]. For example, adolescents who use tobacco/smoke are seen as antisocial by society, and addicted adolescents are more likely to be isolated and rejected by their peers, leading to loneliness [79]. Moreover, loneliness is a part of depression, and previous studies stated that sedentary behavior might result in depressive mode [30, 80]. In addition, adolescents with sedentary behaviors were more likely to use and become addicted to social media, possibly resulting in social isolation [81].

### Strengths and limitations of the study

The study had a few limitations. First, because the study was cross-sectional, it was impossible to establish causal associations between adolescent loneliness trajectories and predictor variables. Second, the study’s respondents were only in-school adolescents, which limited our ability to generalize the results to out-of-school adolescents, who may experience different emotional and social environments. Third, the survey employed a self-reporting data collection design, which may lead to over-reporting or under-reporting. Fourth, in the GSHS survey, a single-item question was used to measure loneliness status, excluding help-seeking behaviors and multidimensional aspects related to loneliness. A multi-item loneliness scale would yield more reliable findings. Moreover, perceived loneliness may differ among adolescents with help-seeking behaviors compared to those without. Therefore, future studies (i.e., longitudinal studies) should consider help-seeking behavior and a multi-item measurement of adolescent loneliness. Although listwise deletion simplifies analysis, it may introduce bias if excluded adolescents systematically differ from those included. Hence, future studies could apply multiple imputations or other robust approache to address missing data more comprehensively. Lastly, using older data was an important limitation; however, these are the most recent nationally representative and comparable datasets available for the countries included in this study. The standardized methodology and the high response rates of the GSHS data represent a valuable source for capturing adolescent health in South Asia, where more recent national surveillance data are limited. Therefore, the findings of this study should be interpreted as an important baseline for understanding mental health patterns of adolescents in South Asia, rather than as current prevalence estimates.

## Conclusion

The study found that nearly one in six girls and one in nine boys reported loneliness in Afghanistan, Bangladesh, and Pakistan, with the highest prevalence in Afghanistan. Several risk factors were identified, including lack of close friends, anxiety-induced sleep disturbances, suicidal ideation, bullying, food insecurity, passive smoking, and sedentary behavior. These findings highlight the need for targeted interventions to address loneliness among in-school adolescents in South Asian countries. Recommendations may include promoting peer and parental support, social skills programs, school-based counseling services, and addressing food insecurity. These efforts align with Sustainable Development Goal 3.4, which focuses on mental health and well-being.

## Supplementary Material

Supplementary Material 1.

## Data Availability

Data associated with this study have been deposited at WHO and can be downloaded from [https://extranet.who.int/ncdsmicrodata/index.php/catalog/GSHS](https:/extranet.who.int/ncdsmicrodata/index.php/catalog/GSHS) .
