# Supplementary material for: Gender and spatial variation of loneliness among adolescents in three South Asian countries: prevalence and its correlates
Source: BMC Public Health. 2026 Jan 27;26:669. doi: 10.1186/s12889-025-26069-7 (PMC12918254; doi:10.1186/s12889-025-26069-7)
Supplement: Supplementary file 1 — Supplementary Material 1. [file 12889_2025_26069_MOESM1_ESM.docx]

**Appendix**

**Table A1:** Description of the study variables

| **Variables** | **Question** | **Response options (coding scheme)** |
| --- | --- | --- |
| **Outcome variable** | | |
| Loneliness | “During the past 12 months, how often have you felt lonely?” | “1=never to 5=always (coded 1–3=0 and 4–5=1)” |
| **Control variables** | | |
| Age | “How old are you?” | “11 years old or younger to 18 years old or older” |
| Sex | “What is your sex?” | “Male, Female” |
| Hunger | “During the past 30 days, how often did you go hungry because there was not enough food in your home?” | “1 = never to 5 = always (coded 1=0, 2–3=1, and 4–5=2)” |
| Peer support | “During the past 30 days, how often were most of the students in your school kind and helpful?” | “1 = never to 5 = always (coded 1–2=0, 3=1, and 4–5=1)” |
| Parental supervision | “During the past 30 days, how often did your parents or guardians check to see if your homework was done?” | “1=never to 5=always (coded 1–3=0 and 4–5=1)” |
| Parental connectedness | “During the past 30 days, how often did your parents or guardians understand your problems and worries?” | “1=never to 5=always (coded 1–3=0 and 4–5=1)” |
| Parental bonding | “During the past 30 days, how often did your parents or guardians really know what you were doing with your free time?” | “1=never to 5=always (coded 1–3=0 and 4–5=1)” |
| **Explanatory Variables** | | |
| ***Poor mental health outcomes*** | | |
| No close friends | “How many close friends do you have?” | “1 = 0 to 4 = 3 or more (coded 1+=0, 0=1)” |
| Anxiety | “During the past 12 months, how often have you been so worried about something that you could not sleep at night?” | “1=never to 5=always (coded 1–3=0 and 4–5=1)” |
| Suicide ideation | “During the past 12 months, did you ever seriously consider attempting suicide?” | “0= no, 1= yes” |
| Suicide plan | “During the past 12 months, did you make a plan about how you would attempt suicide?” | “0= no, 1= yes” |
| ***Social–environmental outcomes*** | | |
| Bullied | “During the past 30 days, on how many days were you bullied?” | “1=0 days to 7=All 30 days (coded 1=0, 2–7=1)” |
| Physically attacked | “During the past 12 months, how many times were you physically attacked?” | “1=0 times to 8=12 or more times (coded 1=0, 2–8=1)” |
| Physically fight | “During the past 12 months, how many times were you in a physical fight?” | “1=0 times to 8=12 or more times (coded 1=0, 2–8=1)” |
| Passive smoking | “During the past 7 days, on how many days have people smoked in your presence?” | “1=0 days to 5=all 7 days (coded 1=0, 2–5=1)” |
| School truancy | “During the past 30 days, on how many days did you miss classes or school without permission?” | “1=0 days to 5= 10 or more days (coded 1=0, 2–5=1)” |
| ***Health risk behaviors outcomes*** | | |
| Current smoking | During the past 30 days, on how many days did you smoke at least one cigarette? | “1=0 days to 7=All 30 days (coded 1=0 and 2–7=1)” |
| Current tobacco use | “During the past 30 days, on how many days did you use any tobacco products other than cigarettes, such as biri, jarda, tobacco leaf, gul, or shisha?” | “1=0 days to 7=All 30 days (coded 1=0 and 2–7=1)” |
| Physical inactivity | “Physical activity is any activity that increases your heart rate and makes you get out of breath some of the time. Physical activity can be done in sports, playing with friends, or walking to school. Some examples of physical activity are running, fast walking, biking, dancing, football, swimming, baseball, and cross–fit. During the past 7 days, on how many days were you physically active for a total of at least 60 minutes per day?” | “0=0 days to 7=7 days (coded 1–7=0 and 8=1)” |
| Leisure time sedentary behavior | “How much time do you spend during a typical or usual day sitting and watching television, playing computer games, talking with friends, or doing other sitting activities, such as using the computer or cell phone?” | “1 = Less than 1 hour per day to 6 = 8 or more hours a day (coded 1-2 = 0 and 3-6=1)” |
| Fast food consumption | “During the past 7 days, on how many days did you eat food from a fast food restaurant, such as country-specific examples?” | “1=0 days to 8=7 days (coded 1–2=0, 3–8=1)” |
| Soft drink intake | “During the past 30 days, how many times per day did you usually drink carbonated soft drinks, such as country-specific examples? (Do not include diet soft drinks.)” | “1=0 times to 7=5 or more times (coded 1–4=0, 5–7=1)” |
| Fruits intake | “During the past 30 days, how many times per day did you usually eat fruit such as country-specific examples?” | “1 = I did not eat fruit during the past 30 days  to 7 = 5 or more times per day” |
| Vegetable intake | “During the past 30 days, how many times per day did you usually eat vegetables, such as country-specific examples?” | “1=I did not eat vegetable during the past 30 days to 7=5 or more times per day” |
